# Patient With Lipoid Proteinosis Presenting With Sudden-Onset Lower Limb Weakness: A Rare Case

**DOI:** 10.7759/cureus.69018

**Published:** 2024-09-09

**Authors:** Hassan Mobarak, Sandra El Helou

**Affiliations:** 1 Psychiatry and Behavioral Sciences, Lebanese American University School of Medicine, Beirut, LBN; 2 Radiology, Lebanese American University School of Medicine, Beirut, LBN

**Keywords:** acute ischemia, ct and mri brain, dermatological manifestations, lipoid proteinosis (lp), neurological deficit

## Abstract

Lipoid proteinosis (LP), or Urbach-Wiethe disease, is an infrequent autosomal recessive disorder typified by hyaline material deposition in diverse tissues, including the skin, mucous membranes, and internal organs. This case report addresses an unusual presentation of LP in a 25-year-old male, whose initial symptom was sudden-onset left lower limb weakness. This deviation from the typical dermatological and laryngeal manifestations prevalent in LP compels us to consider LP as a potential causative factor in neurological deficits.

Physical examination revealed motor weakness in the left lower limb with several dermatological manifestations. Laboratory tests indicated potential thyroid, liver, and urinary tract pathologies. Brain imaging studies revealed bilateral mesial temporal lobe calcifications consistent with LP with associated cortico-subcortical infarcts and hemorrhagic transformation. Additionally, foraminal disc protrusion at L5-S1 in the patient's back MRI suggested nerve compression contributing to limb weakness.

This patient's sudden-onset left lower limb weakness accompanied by imaging findings of cortical and subcortical abnormalities aligns with the spectrum of neurological manifestations associated with LP but is less commonly reported. Moreover, the disturbed liver and thyroid blood tests in this patient suggest a potential link between LP and thyroid and liver pathologies, which needs further investigation.

This case illustrates that motor weakness, potentially due to cerebral infarcts and intracerebral hemorrhage, is a possible complication of LP. Further research is necessary to confirm and understand the phenomenology of this complication.

## Introduction

Lipoid proteinosis (LP), also known as Urbach-Wiethe disease, is a rare autosomal recessive disorder characterized by hyaline material deposition in various tissues [1,2]. It predominantly affects the skin, mucous membranes, and internal organs. Although the classical presentation found in almost all patients is a weak cry and a persistent hoarse voice during the first years of life, clinical manifestations exhibit wide heterogeneity, including dermatological and neurological symptoms [3].

Oral features are common findings in patients with LP, with the tongue most frequently affected, followed by the floor of the mouth, lips, and buccal mucosa [4]. Neurological complications are noted in a subset of patients, manifested as cognitive impairment, behavioral abnormalities, motor deficits, and epilepsy, which are found in 30% of cases [5-9]. Nevertheless, the onset of sudden lower limb weakness as an initial symptom in LP is extraordinarily rare, with few reports documented in the literature.

This report highlights a case of a young male patient (mid-20s), previously diagnosed with LP, who presented with sudden-onset left lower limb weakness. The purpose of this report is to underscore the need to acknowledge and regard LP as a potential causative factor in patients manifesting neurological deficits, even in the absence of characteristic cutaneous or laryngeal features. By shedding light on this rare case, we aim to augment the existing LP literature and raise awareness about its diverse clinical presentations, especially regarding neurological involvement. Understanding LP's broad manifestation spectrum is imperative for timely diagnosis, appropriate management, and improved patient outcomes.

## Case presentation

A 25-year-old male patient with a medical history of LP and hypothyroidism presented with sudden-onset left lower limb weakness that had been ongoing for four days. This was associated with urinary incontinence and a progressively worsening aphasia. The patient had exhibited limping for the past two weeks due to a painful bulla on the right foot's plantar surface. The family reported no incidences of head trauma or falls but mentioned non-mucoid and non-bloody diarrhea over the past three months.

**Figure 1:**
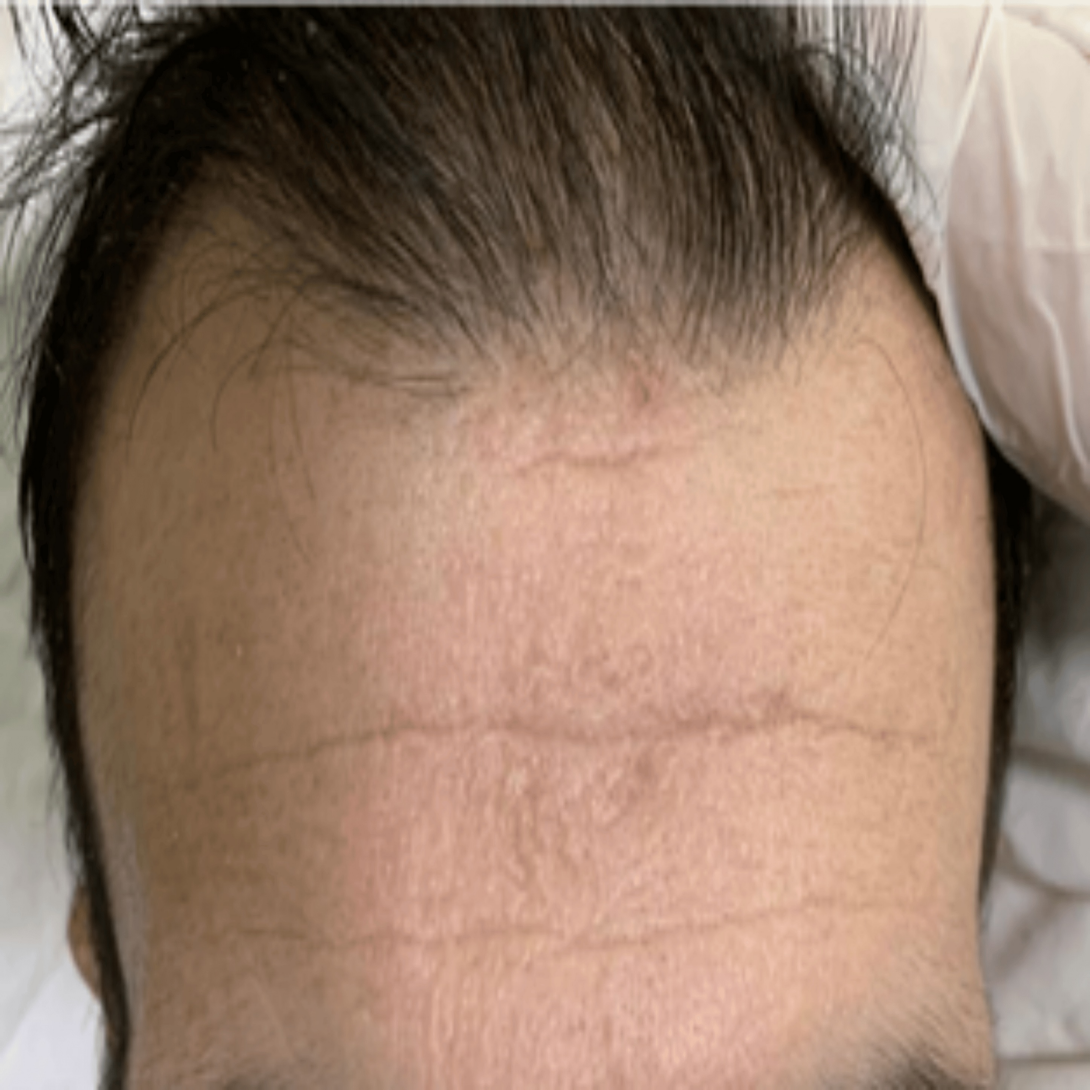
Clinical manifestations of LP: waxy forehead with atrophic depressed scars LP: lipoid proteinosis

**Figure 2:**
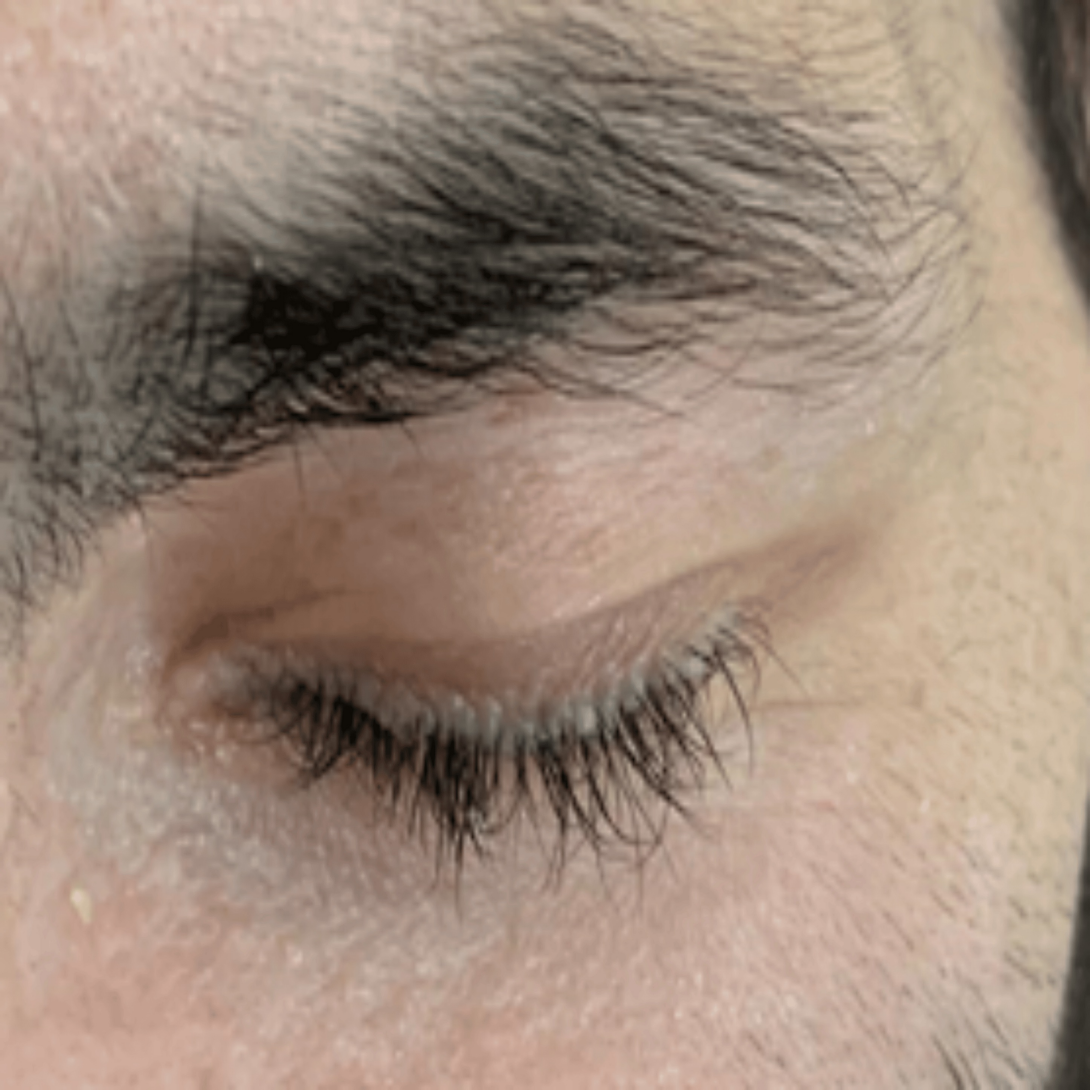
Clinical manifestations of LP: row of beaded papules (moniliform blepharosis) along the eyelid margins LP: lipoid proteinosis

**Figure 3:**
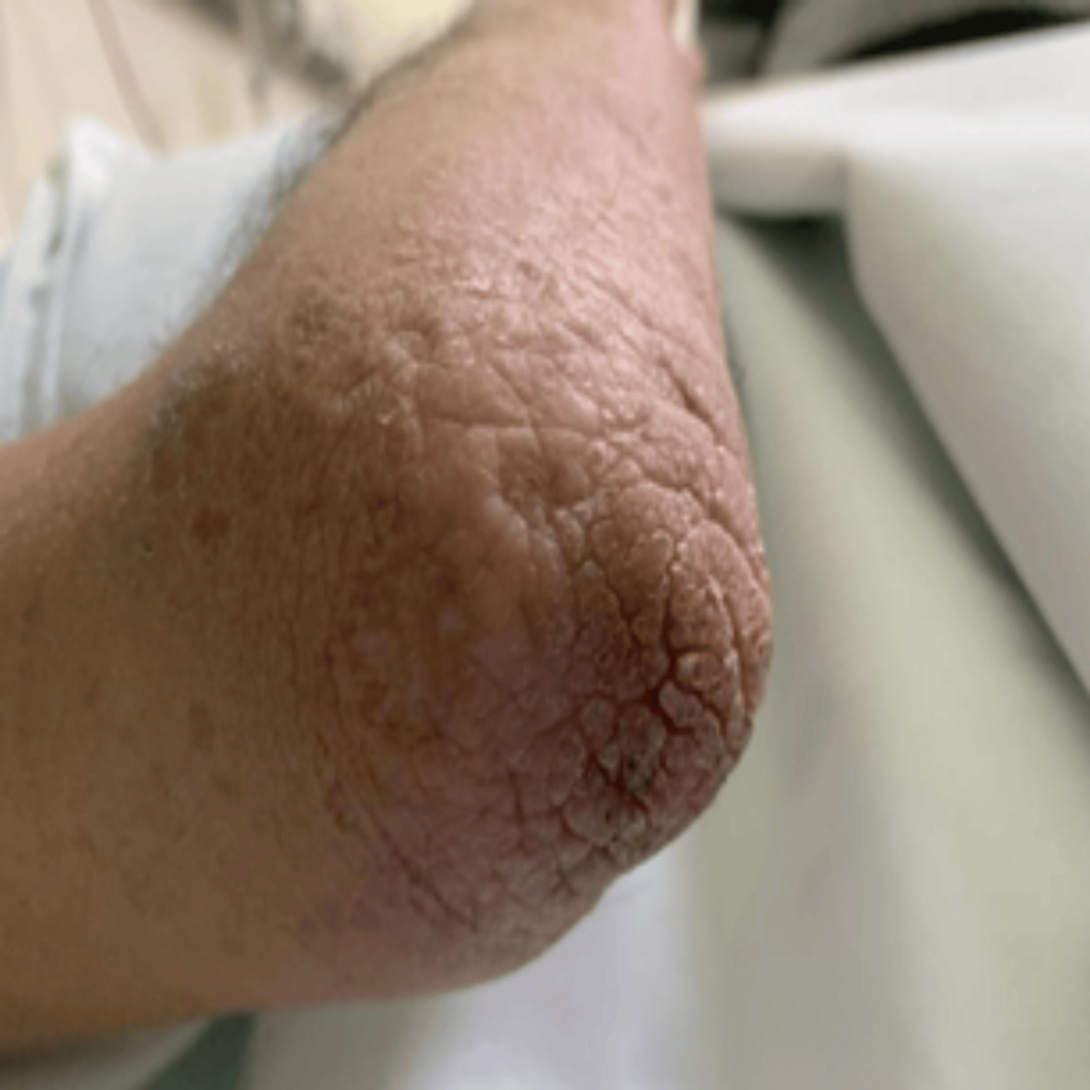
Clinical manifestations of LP: hyperkeratotic, verrucous plaques on the elbow LP: lipoid proteinosis

**Figure 4:**
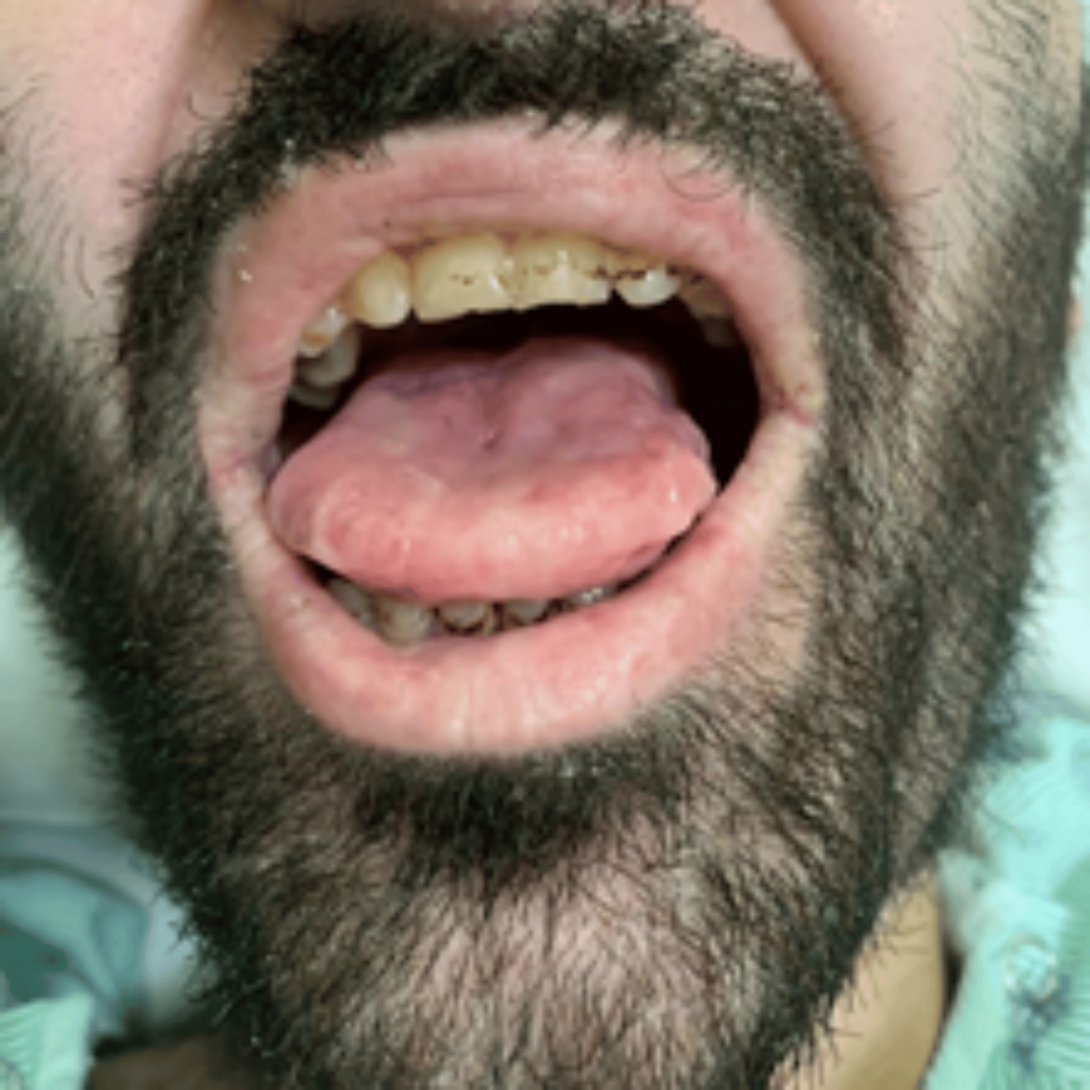
Clinical manifestations of LP: firm and woody tongue with ulceration and a cobblestone appearance LP: lipoid proteinosis

**Figure 5:**
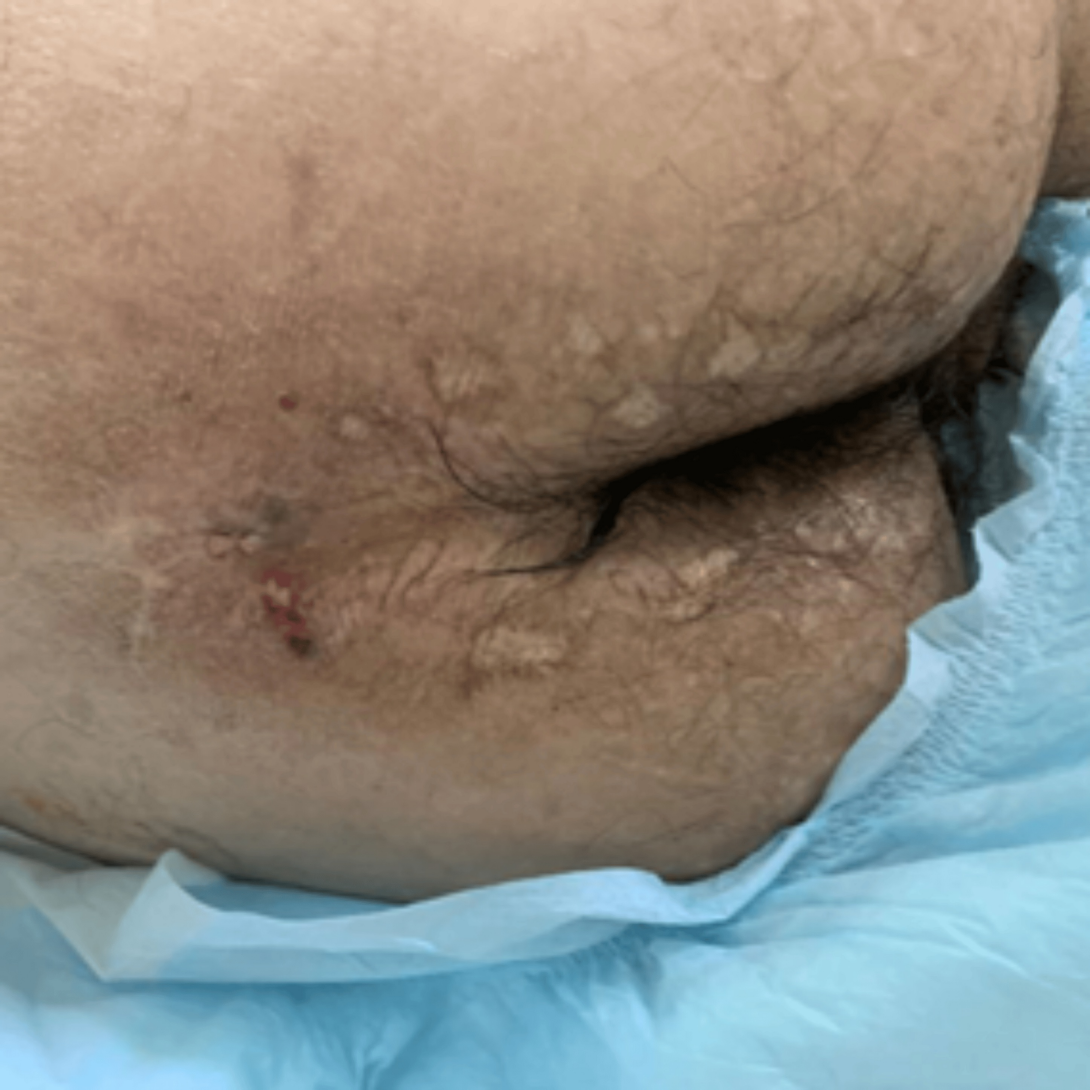
Clinical manifestations of LP: scarring from previously healed bullae in the perianal area LP: lipoid proteinosis

**Figure 6:**
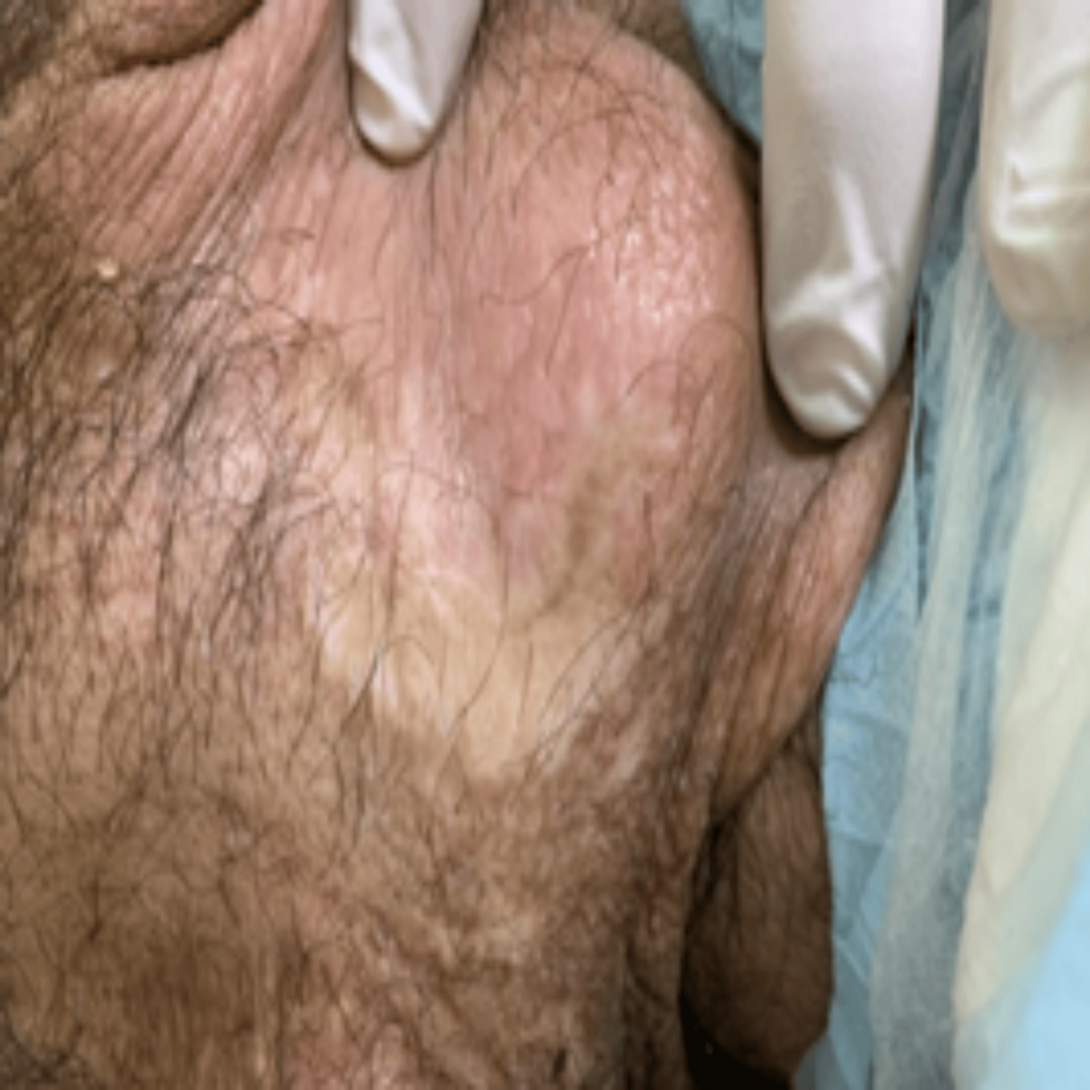
Clinical manifestations of LP: scarring from previously healed bullae in the scrotum LP: lipoid proteinosis

**Figure 7:**
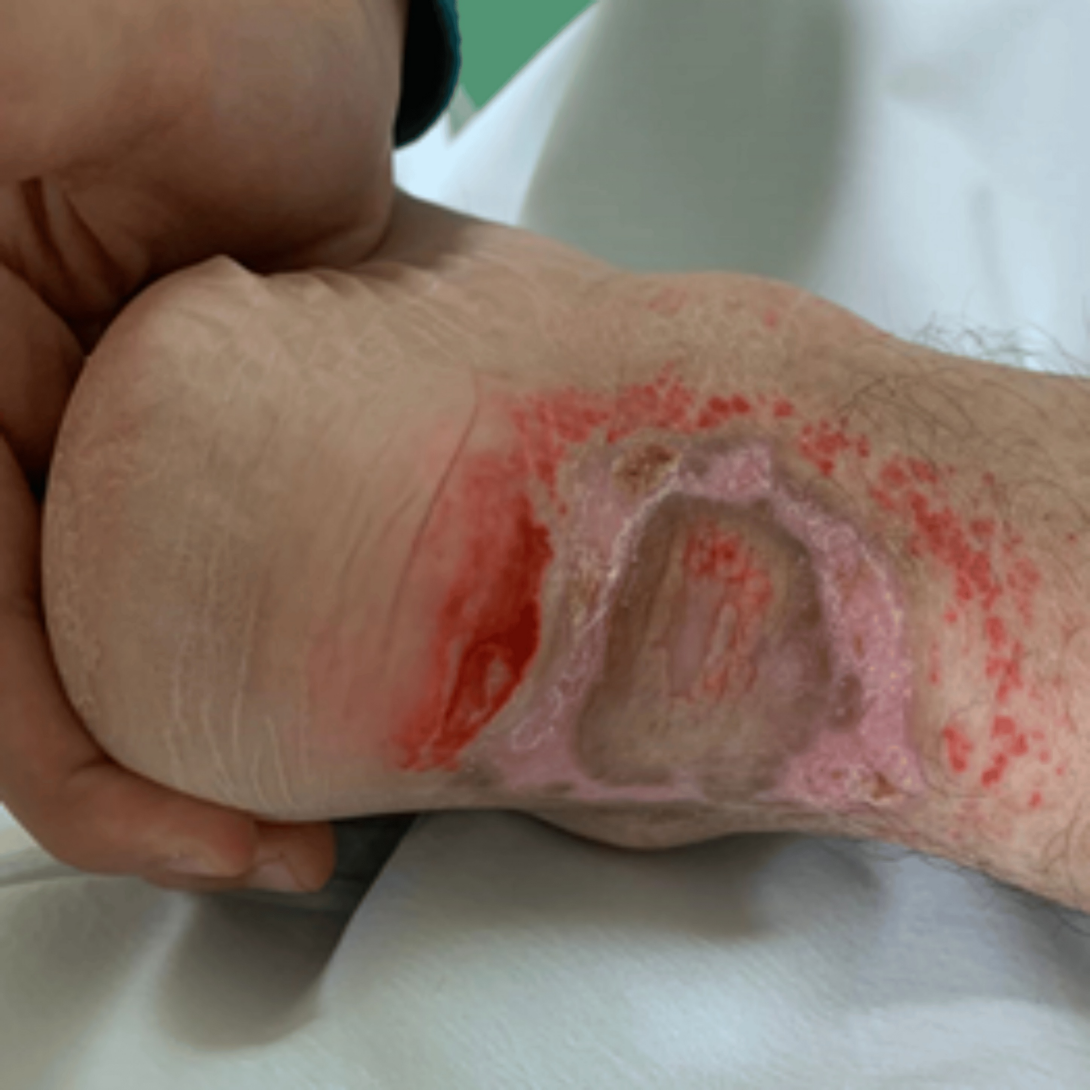
Clinical manifestations of LP: scarring from previously healed bullae at the right ankle's posterior surface LP: lipoid proteinosis

Physical examination revealed a motor power of 0/5 in the left lower limb, 4/5 in the left upper limb, and 5/5 in the right extremities. The patient had a history of a hoarse voice since childhood and appeared underweight and pale. Several dermatological manifestations were noted, including a waxy forehead with atrophic depressed scars (Figure 1), diffuse hair loss characterized by thin, poor-quality hair, and a row of beaded papules or moniliform blepharosis along the eyelid margins (Figure 2). Hyperkeratotic, verrucous plaques were observed on the patient's elbows (Figure 3). The patient's tongue was firm and woody, displayed ulceration and a “cobblestone” appearance, and had limited protrusion due to frenulum infiltration (Figure 4). Scarring from previously healed bullae was evident in the perianal area, scrotum, and on the right ankle's posterior surface (Figures 5-7).

Blood tests included an increased white blood cell (WBC) count, increased thyroid-stimulating hormone (TSH), increased γ-glutamyl transferase (GGT), normal creatinine, decreased hemoglobin, increased procalcitonin, and a normal international normalized ratio (INR) (Table 1).

**Table 1:** Patient's blood test

Blood tests	Patient’s values	Normal values
White blood cell count (WBC)	58.8 x 10^9^/L	4.0-10.0 x 10^9^/L
Thyroid-stimulating hormone (TSH)	11.72 µU/mL	0.5-5.0 µU/mL
γ-glutamyl transferase (GGT)	503 units/L	0-30 units/L
Creatinine	0.5 mg/dL	0.7-1.3 mg/dL
Hemoglobin	11.9 g/dL	14-18 g/dL
Procalcitonin	0.33 ng/ml	<0.1 ng/ml
International normalized ratio (INR)	1.36	<1.5

**Figure 8:**
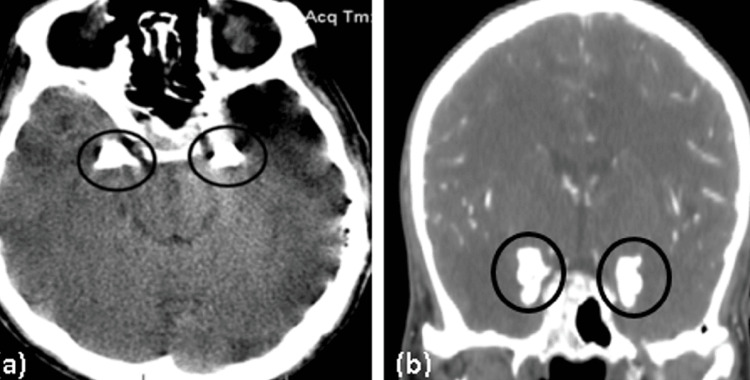
Axial non-enhanced (a) and coronal enhanced CT scan of the brain (b) demonstrating bilateral calcifications at the mesial temporal lobes (black circles) CT: computed tomography

**Figure 9:**
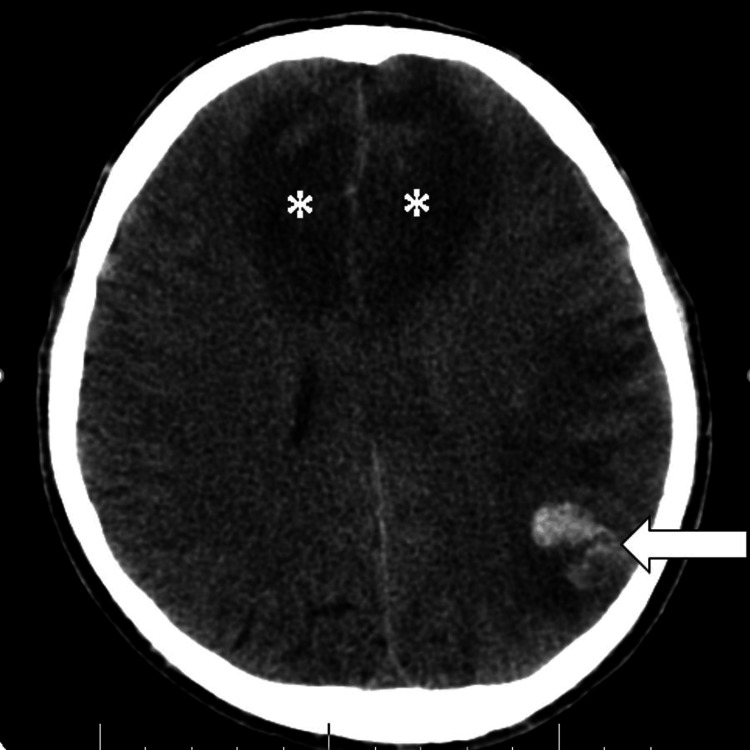
Axial non-enhanced CT scan of the brain. Left posterior parietal intraparenchymal hemorrhage and surrounding edema (white arrow), with bilateral frontal cortico-subcortical hypodensities consistent with cytotoxic edema (asterisks) CT: computed tomography

Urine analysis showed the presence of numerous red blood cells (RBC): 10-12 RBCs/high power field (hpf). Stool analysis did not reveal any abnormalities. Serology testing for HIV and toxoplasma indicated no acute or chronic infection. A brain CT scan initially revealed bilateral mesial temporal lobe calcifications consistent with LP (Figure 8). The scan also depicted left posterior-parietal cortical intraparenchymal hemorrhage with surrounding edema, as well as bilateral anterior frontal cortico-subcortical hypodensities consistent with cytotoxic edema (Figure 9).

**Figure 10:**
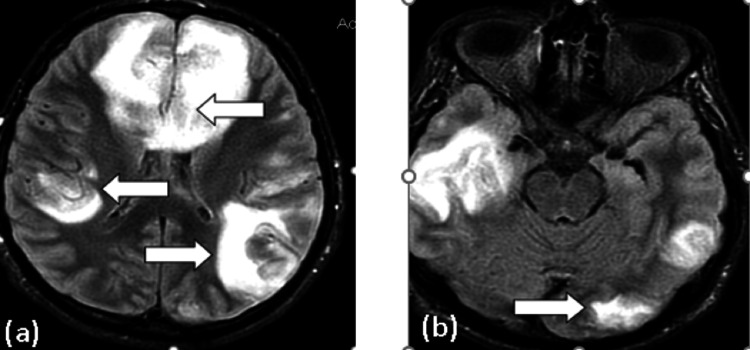
Axial non-enhanced MRI of the brain FLAIR sequences. Bilateral confluent and nodular cortex in the fronto-parieto-temporal (a) and left occipital lobes (b), accompanied by surrounding edema (arrows) MRI: magnetic resonance imaging, FLAIR: fluid-attenuated inversion recovery

**Figure 11:**
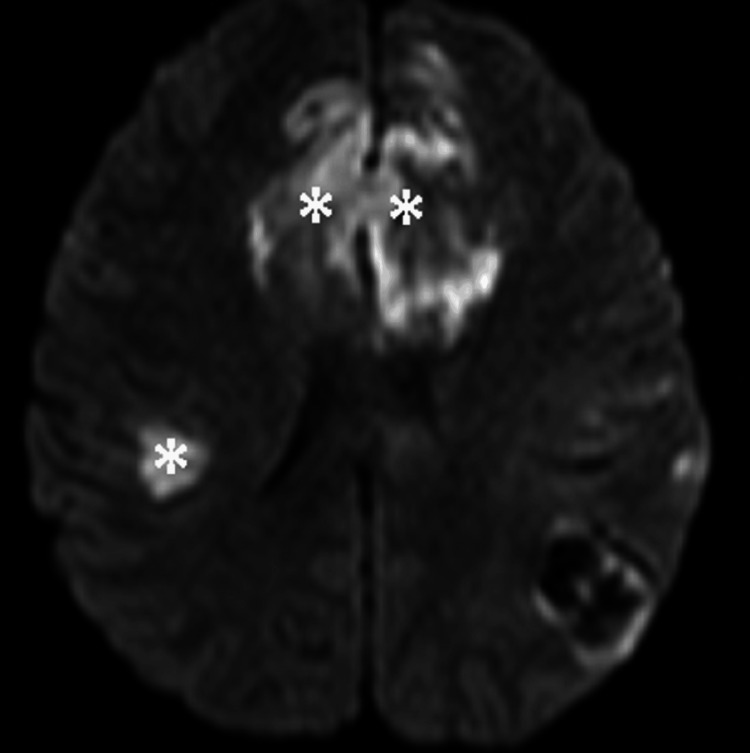
Axial non-enhanced MRI of the brain. Diffusion-weighted image shows areas of high signal and low ADC (not shown) involving the bi-frontal and right parietal lobes (asterisks) consistent with infarcts MRI: magnetic resonance imaging, ADC: apparent diffusion coefficient

**Figure 12:**
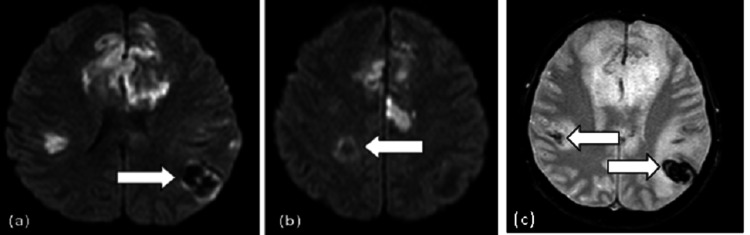
Axial non-enhanced MRI of the brain. Diffusion-weighted images show hypointense foci with a continuous rim of bright signal involving the left parietal (a) and right parietal lobes (b), consistent with hemorrhage (arrows). (c) SWI sequence demonstrates susceptibility artifacts in keeping with blood products MRI: magnetic resonance imaging, SWI: susceptibility weighted imaging

MRI results showed confluent and nodular cortex in the bilateral fronto-parieto-temporal and left occipital lobes, accompanied by surrounding cytotoxic edema (Figure 10). Restricted diffusion was observed in the right parietal and bilateral frontal regions (Figure 11), denoting infarcts, while the left parietal and right parieto-temporal regions exhibited hemorrhagic characteristics (Figure 12).

However, a repeated CT scan with contrast revealed no nodular, ring, homogeneous, or heterogeneous contrast enhancement within the affected regions. An MRI of the patient's back showed slight left-sided scoliosis and straightening of the lumbar lordosis, noting a foraminal disc protrusion at the L5-S1 level, subsequently compressing the nerve root.

## Discussion

The case report presents a young adult man with LP. The patient had a range of symptomatology, including a hoarse voice, atrophic depressed scars, hyperkeratotic plaques, and tongue abnormalities, aligning with the typical cutaneous and mucosal manifestations of LP. Brain imaging showed mesial temporal lobe calcifications, cortico-subcortical infarcts, and intracranial hemorrhage each at specific locations in the brain. Laboratory findings indicated elevated TSH and GGT levels, suggesting potential thyroid and liver pathology. These abnormalities could be due to hypothyroidism and LP-related liver disease, respectively. The detection of numerous RBCs in the urine analysis also suggested possible kidney or urinary tract involvement. The patient's neurological symptoms pose an unusual presentation, which included acute onset lower limb weakness and urinary incontinence.

Neurological involvement in LP can manifest through a variety of symptoms, including epilepsy, dystonia, and spontaneous CNS hemorrhage [10-11]. Neuropsychiatric symptoms are also reported, including memory loss and other cognitive impairments, behavioral changes, hallucinations, and schizophreniform illness [12-14]. This patient's sudden-onset left lower limb weakness and urinary incontinence, accompanied by imaging findings of cortical and subcortical abnormalities, aligns with the spectrum of neurological manifestations associated with LP but is less commonly reported. One case report we found described an episode of hemi-facial weakness rather than extremity weakness [15].

The bilateral mesial temporal lobe calcifications seen on the CT scan are consistent with previous reports [16-20]. These calcifications are typically associated with epilepsy and cognitive deficits [7]. However, the presence of intracranial infarcts and hemorrhagic transformation is an unusual finding and suggests a more complex pathophysiological mechanism.

There were some diagnostic challenges in this case. The initial MRI findings of restricted diffusion and hemorrhagic lesions needed a thorough differential diagnosis. The absence of contrast enhancement on the post-contrast CT scan ruled out cerebral toxoplasmosis and CNS lymphoma, which are often considered in immunocompromised patients presenting with similar imaging features. However, the imaging findings pointed toward acute ischemic infarction with hemorrhagic conversion, a condition that requires immediate and intensive management. Moreover, the MRI of the lumbar spine revealed a foraminal disc protrusion at the L5-S1 level, which could have contributed to the patient's lower limb weakness by compressing the nerve root.

While there is no well-established correlation between hypothyroidism and LP, endocrine abnormalities, including thyroid dysfunction, might be observed in some patients with multisystem diseases like LP. In this case, elevated TSH levels could suggest hypothyroidism, which warrants further investigation to understand any potential link between these conditions. Future studies could explore whether thyroid dysfunction is a coincidental finding or if there is a pathophysiological connection with LP.

This case highlights the need for a comprehensive and multidisciplinary approach to the evaluation and management of patients with LP, particularly those presenting with atypical symptoms. The interplay between LP, potential endocrine abnormalities, and neurological complications necessitates a thorough workup to identify and address all contributing factors. This approach is crucial for improving patient outcomes and preventing misdiagnosis or delayed diagnosis.

This case report also highlights the need for further research into the neurological and endocrine manifestations of LP. Genetic testing for extracellular matrix protein 1 (ECM1) mutations can provide definitive confirmation of the diagnosis. Longitudinal studies focusing on the neurological aspects of LP are essential for understanding the disease's evolution and developing targeted therapeutic approaches.

Additionally, the observation of elevated TSH and GGT levels in this patient suggests a potential link between LP and thyroid and liver pathologies, which needs further investigation. Future studies should explore the prevalence and impact of these comorbidities in LP patients to enhance the understanding and management of this rare disorder.

## Conclusions

Although LP predominantly affects the skin, it can also involve internal organs and the CNS, leading to a wide range of symptoms, including neurological and psychiatric manifestations. This case illustrates that motor weakness, potentially due to cerebral infarcts and intracerebral hemorrhage, is a possible complication of LP. Further research is necessary to confirm and understand the phenomenology of this complication, particularly its distinctive findings on brain imaging. Expanding the differential diagnosis of sudden-onset weakness to include LP-related complications can lead to improved patient care and outcomes.
